# Return on Investment of the Back At Work After Surgery (BAAS) Care Pathway Compared to Care-as-Usual in Knee Arthroplasty

**DOI:** 10.1007/s10926-025-10328-w

**Published:** 2025-09-19

**Authors:** Daniël O. Strijbos, A. Carlien Straat, Geert van der Sluis, Wim F. C. van Houtert, Johanna M. van Dongen, Tim Boymans, P. Paul F. M. Kuijer, Michiel F. Reneman

**Affiliations:** 1 Department of Public and Occupational Health, Amsterdam UMC, University of Amsterdam, Meibergdreef 9, 1105 AZ Amsterdam, The Netherlands; 2 Department of Health Innovations, Nij Smellinghe Hospital Drachten, Compagnonsplein 1, 9202 NN Drachten, The Netherlands; 3 Hanze University of Applied Sciences Groningen, Zernikeplein 7, 9747 AS Groningen, The Netherlands; 4 Department of Health Sciences & EMGO+ Institute for Health and Care Research, Faculty of Earth & Life Sciences, VU University Amsterdam, De Boelelaan 1085, 1081 HV Amsterdam, The Netherlands; 5 Department of Orthopaedics, Maastricht UMC +, P. Debyelaan 25, 6229 HX Maastricht, The Netherlands; 6 Department of Rehabilitation, University Medical Center Groningen, University of Groningen, Groningen, The Netherlands

**Keywords:** Return on investment, Orthopedic procedures, Rehabilitation, Occupational medicine, Knee, Arthrosis

## Abstract

**Purpose:**

The Back At work After Surgery (BAAS) care pathway integrates medical and occupational care to enhance return to work (RTW) after knee arthroplasty (KA). BAAS has shown effectiveness in improving RTW outcomes, but its economic impact is unknown. This study evaluates the costs and return on investment (ROI) of BAAS compared with care-as-usual.

**Methods:**

This multicenter, prospective cohort study involved 270 employed patients having KA, comparing the BAAS pathway (*n* = 137) to care-as-usual (ACTIVE trial, *n* = 133). The ROI was evaluated from societal and employer’s perspectives. Productivity (absenteeism and presenteeism) and healthcare (primary and secondary) costs were assessed using cost questionnaires administered at 3, 6, 9, and 12 months post-surgery. Propensity score matching and multiple imputation addressed non-randomization and missing data, respectively. ROI was calculated by dividing the netto benefits—defined as reductions in productivity and healthcare costs, or productivity costs alone minus costs of the BAAS intervention—by the intervention costs multiplied by 100%.

**Results:**

Propensity score-matched analyses included 102 patients per cohort. The total netto benefits from employers and societal perspective were of €4,493 and €4,982, respectively. Intervention costs were €845/patient. This resulted in a ROI of 590% (95% CI 67–1112%) and 532% (95% CI 27–1037%) from the societal and employer’s perspective, respectively, per Euro invested.

**Conclusions:**

The BAAS care pathway demonstrates a favorable economic impact through significant 12 month downstream reductions in absenteeism and healthcare costs, and a positive ROI from both the societal and employer perspective.

*Trail registration***:** This study was retrospectively registered at clinicaltrails.gov (https://clinicaltrials.gov/ct2/show/NCT05690347, date of first registration: 19-01-2023).

**Supplementary Information:**

The online version contains supplementary material available at 10.1007/s10926-025-10328-w.

## Introduction

Work participation is a key determinant of individual health and well-being, yet it is often overlooked in clinical practice and research [1–6]. Return to work (RTW) after surgery is particularly relevant for individuals having knee arthroplasty (KA), as the incidence of KA is rising, especially among working-age patients [7–11]. Despite favorable outcomes in terms of pain relief and knee function, RTW rates following KA remain suboptimal, with global non-RTW rates reaching 35% [12]. In the Netherlands, approximately 31% of patients do not RTW after KA, contributing to a substantial socioeconomic burden [13]. The average annual cost of sick leave related to knee osteoarthritis in the Dutch workforce has been estimated at €26.9 million, underscoring the need for effective interventions that facilitate timely and sustained RTW [14].

Traditionally, postoperative care pathways for KA have prioritized medical and functional recovery, with limited attention to occupational reintegration. However, growing evidence suggests that integrating medical and occupational care can enhance RTW outcomes [15–20]. Interdisciplinary multimodal approaches, with coordinated modalities, such as rehabilitation, occupational support, and personalized guidance, have demonstrated effectiveness in reducing sick leave duration in other musculoskeletal conditions, but not surrounding KA. In response to this gap in KA care, the Back At work After Surgery (BAAS) clinical pathway was developed and tested on feasibility [21, 22]. BAAS emphasizes the early and structured integration of medical and occupational care, with a patient-centered approach that actively involves individuals in their RTW process. In previous studies, BAAS has demonstrated feasible and effectiveness on RTW outcomes without compromising clinical outcomes: more patients (98% vs. 87–84%) started RTW earlier (16–25 days) and did fully RTW earlier (27 days). However, its economic impact remained unexamined [20].

This study aimed to evaluate the costs and return on investment (ROI) of the BAAS care pathway compared with care-as-usual from both the societal and employer’s perspective. By addressing a critical economic gap in KA care and rehabilitation, this study may provide financial support for work-integrated perioperative care that could be extended to other surgical populations if proven economic viable.

## Method

### Study Design

We conducted a multicenter prospective cohort study evaluating the costs and ROI of BAAS compared to a matching care-as-usual cohort from the ACTIVE trial [22, 23]. Ethical approval for the study was granted (reference IDs: 108 W21_454#21.504 and L1429.2021). This economic evaluation is reported according to the Consolidated Health Economic Reporting Standards 2022 (CHEERS 2022) [24].

### Study Population, Setting, and Location

Inclusion and exclusion criteria were similar for both cohorts (Table 1). The recruitment procedure for BAAS is described in the protocol paper [22]. The BAAS study was performed in two hospitals in the Netherlands, Nij Smellinghe Hospital (NS) and Elizabeth Tweesteden Ziekenhuis (ETZ). These hospitals perform approximately 450 and 600 KAs annually. The patients in the care-as-usual cohort from the ACTIVE trial were recruited in eleven Dutch clinics and hospitals, performing 100–800 KAs annually [23].

**Table 1:** Inclusion and exclusion criteria, outcomes, and covariates of the BAAS intervention and ACTIVE care-as-usual cohorts

Cohort	Back At work After Surgery intervention	ACTIVE care-as-usual
Inclusion	Nov. 2021 to Apr. 2023	Oct. 2020 to Jan. 2023
Follow-up	Twelve months	Twelve months
Inclusion criteria	1. Primary UKA or TKA 2. Age 18–65 years 3. Paid work for at least 8 h/week 4. Intention to RTW	1. Primary UKA or TKA 2. Age 18–67 years 3. Paid work for at least 8 h/week 4. Intention to RTW
Exclusion criteria	1. Receiving more than one medical event affecting workability within one year 2. KA for reasons other than knee osteoarthritis 3. Major disabling mental disorders 4. Not returning any of the sent cost questionnaire	1. Another planned joint replacement during the study period 2. Extreme comorbidity affecting recovery 3. Not returning any of the sent cost questionnaire
Primary outcomes and covariates		
Primary outcomes	The costs of BAAS intervention Productivity loss (absenteeism and presenteeism) Healthcare (primary and secondary)	The costs of Productivity loss (absenteeism and presenteeism) Healthcare (primary and secondary)
Covariates	1. Sex 2. BMI 3. Type of surgery 4. Knee-straining job 5. Sick leave 6. Work relatedness of KA	1. Sex 2. BMI 3. Type of surgery 4. Knee-straining job 5. Sick leave 6. Work relatedness of KA

### BAAS Intervention

The BAAS intervention is extensively described in our protocol paper [22]. To incorporate patient and public involvement, patients who had previously had KA were consulted in the early development of the BAAS care pathway and assessed the feasibility of the intervention [21]. The BAAS care pathway started with a preoperative consultation by a hospital-based physical therapist. The patients were counseled for optimal preparation before surgery. Also, the patients completed several standardized questionnaires and performed functional tests to set a baseline measurement, which were used for evaluating clinical recovery. A work-related therapy goal was set using Goal Attainment Scaling (GAS). Patients were asked to wear an accelerometer from 2 weeks preoperatively until full RTW was achieved (PAM 2.0, PAM B.V., Oosterbeek, the Netherlands) to monitor physical activity with real-time feedback accessible to patients and healthcare professionals via the Atris app (Peercode, Geldermalsen, the Netherlands). An occupational assessor compiled a report of beneficial and limiting factors regarding RTW based on a preoperative workplace assessment (Appendix I).

Standard postoperative consultations by the hospital were organized at 6 weeks and every 3 months thereafter, up to 12 months or until full RTW was achieved. During these consultations, recovery was assessed by monitoring the GAS goal, and by using the same questionnaires and functional tests as those at baseline. Around the fourth or fifth week after surgery, an online team meeting was held involving the patient, primary care physical therapist, hospital-based physical therapist, occupational assessor, and occupational physician. This consultation focused on evaluating the recovery progress and to use this information to refine the RTW plan, if necessary.

Postoperative care included targeted exercise therapy by a physical therapist in a primary care setting, adhering to the Royal Dutch Society for Physiotherapy guidelines for knee osteoarthritis [25]. If the hospital-based physical therapist expected that a patient could not RTW within 12 months—based on patient experience, clinical assessments, and accelerometer data—patients were referred for a multidisciplinary rehabilitation assessment in an academic hospital. This assessment could lead to participation in a vocational rehabilitation program.

### Care-as-Usual

Patients receiving care-as-usual followed standard pre- and postoperative procedures following the protocols established by their treating hospital or clinic [26]. According to the Dutch Orthopaedic Association (NOV) guidelines, standard care for KA patients primarily includes surgical implantation of the knee prosthesis. This is preceded by a preoperative consultation conducted by a surgeon or physician assistant and complemented by pharmacological management, such as antibiotics and pain relief medication. After surgery, patients have clinical follow-ups, primarily focusing on wound healing and assessing implant placement. This routine care is fully reimbursed by the Dutch healthcare system. Physical rehabilitation may be provided postoperatively, depending on the patient's individual circumstances and insurance coverage. Additionally, patients typically have access to hospital-provided eHealth tools, such as mobile applications (e.g., Patient Journey App) or websites. These digital resources commonly provide appointment schedules, contact details, guidance on wound care, and sometimes general rehabilitation exercises. Notably, these medical care and eHealth tools do not specifically address guidance on returning to work postoperatively.

Parallel to the medical process, working patients in the Netherlands are legally supported by the Gatekeeper Improvement Act, which mandates early and structured RTW guidance after sick leave. Employers and occupational physicians are required to collaborate on a reintegration plan within six weeks of sick leave. The occupational physician evaluates work capacity and facilitates graded work resumption. In more complex cases, an occupational assessor may be involved to assess job demands, possibilities for workplace adaptations, or options for job transfers, often offered one year after sick leave. However, orthopedic care including rehabilitation and occupational support are typically not well integrated, and RTW is not a standard focus within usual perioperative orthopedic care.

### Measures

#### Outcome

Costs were measured from the societal and employer’s perspective. From the societal perspective, costs included those of the BAAS intervention, healthcare utilization, and productivity losses (absenteeism and presenteeism). When the employer’s perspective was applied, healthcare costs were excluded as—in the Dutch situation—these do not accrue to the employer.

The cost of the BAAS intervention was estimated by collecting detailed data on its soft- and hardware costs as well as the time investments of the intervention providers, and valuing them using Dutch standard costs as provided by the Dutch Manual of Costing [27]. The other cost categories were assessed in both cohorts using cost questionnaires administered at 3, 6, 9, and 12 months of follow-up. Absenteeism and presenteeism were measured using iMTA Productivity Cost Questionnaire (iPCQ) -based questions, valued using the average hourly cost of labor in the Netherlands and the human capital approach [28]. The latter means that productivity losses due to absenteeism and presenteeism were valued based on the total number of hours not worked, multiplied by the average hourly wage. Healthcare utilization consisted of the use of primary care (e.g., physical therapist, general practitioner) and secondary care (e.g., hospital stay, medical specialist). Primary and secondary care were valued using Dutch standard costs as provided by the Dutch Manual of Costing, and if unavailable, prices of professional organizations. Costs were expressed in Euros and 2023 was used as reference year.

#### Demographics

Data on age, sex, BMI, type of surgery, and comorbidities were obtained from the electronic medical patient file. Working hours per week, physical demands of the job, preoperative sick leave, being breadwinner, level of education, and being self-employed were asked to the patient in the preoperative consultation.

### Statistical Methods

To account for missing data and the non-randomized nature of the study, analyses were conducted using multiple imputation and nearest neighbor matching based on propensity scores, respectively. First, missing data in cost outcomes and relevant covariates were handled using Predictive Mean Matching (PMM) via the mice package in R. Five imputed datasets were created. Next, propensity scores were estimated for all patients in both cohorts using logistic regression based on prognostic factors for RTW. The latter was done because cost differences were mainly expected to arise from absenteeism and presenteeism. The following covariates were included: sex (female vs. male), type of surgery (UKA vs. TKA), BMI, physical job demands (knee straining vs. non-knee straining), preoperative sick leave (yes vs. no), and the self-reported work relatedness of the knee complaint (ranging from “totally agree” to “totally disagree”). These propensity scores were used to perform nearest neighbor matching with a caliper width of 0.1, ensuring sufficient balance between groups. We evaluated the quality of the matching procedure by visualizing with propensity score density plots, using standardized mean differences and variance ratios, with thresholds of < 0.1 and < 2, respectively. After matching, differences in cost outcomes between groups were tested using paired t-tests appropriate for matched data. Such a parametric frequentist approach was chosen, as research shows that even when data are heavily skewed—accurate standard errors can be obtained when using parametric frequentist approaches in datasets with moderate to large sample sizes [29]. Then, the ROI of the intervention was calculated as

$${\text{ROI}} = \frac{{{\text{Netto }}\;{\text{benefits}}}}{{{\text{Costs}}}}*100\% ,$$

where Netto benefits were defined as reductions in productivity and healthcare costs (societal perspective) or productivity costs alone (employer’s perspective) minus costs of the BAAS intervention, and Costs as the costs of the BAAS intervention. Please note that, in contrast to cost differences, positive Netto benefits indicate reduced spending. The ROI indicates the percentage of profit per Euro invested in the BAAS intervention, with positive ROIs indicating reduced spending [30, 31]. Economic analyses including ROI are calculated over a period of 12 months after surgery. The script used in R is presented in Appendix II.

### Sensitivity Analysis

Three sensitivity analyses were conducted; 1) applying Classification and Regression Tree (CART) instead of Predictive Mean Matching (PMM) for multiple imputation, 2) using a less restrictive matching strategy by increasing the caliper from 0.1 to 0.2, thereby accepting a less optimal match and 3) applying the statistical method on the complete cases without imputation.

## Results

**Fig. 1:**
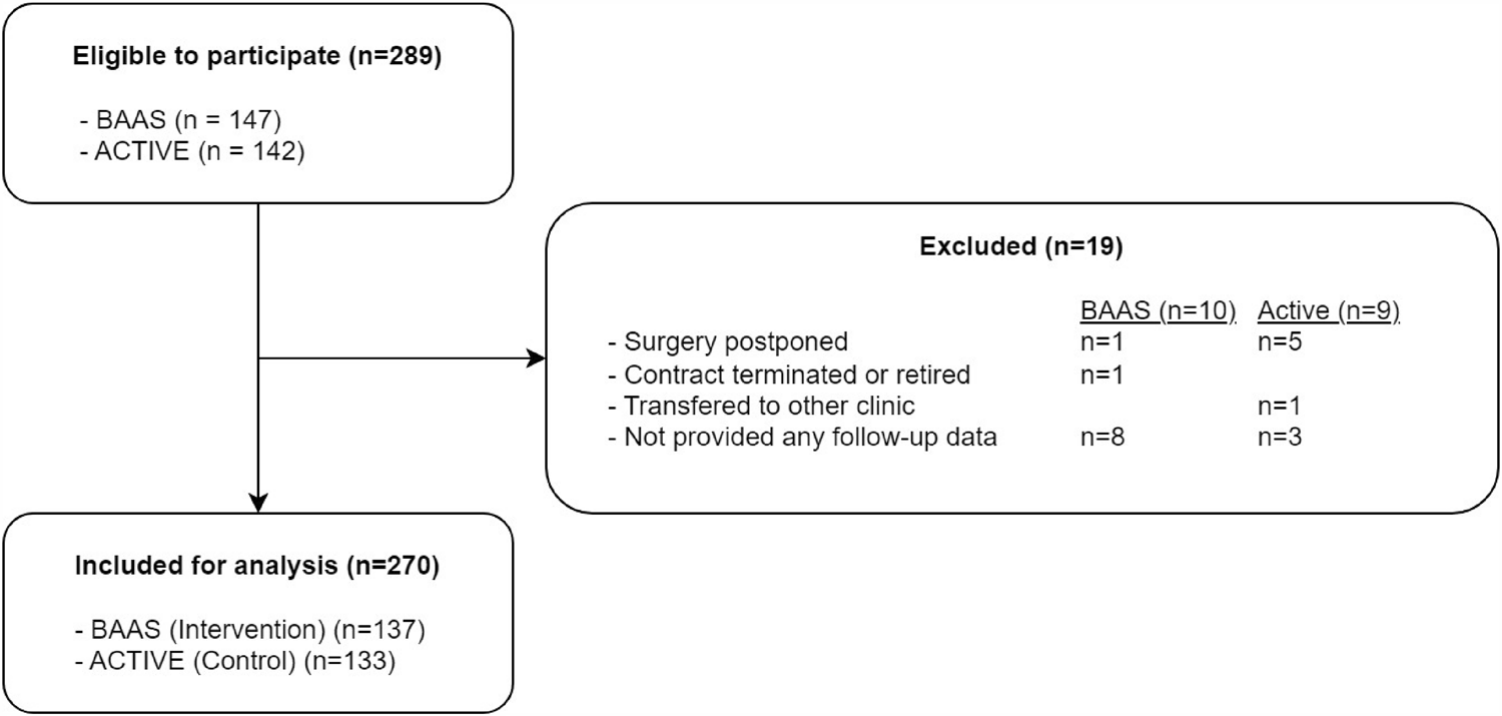
Flowchart

**Fig. 2:**
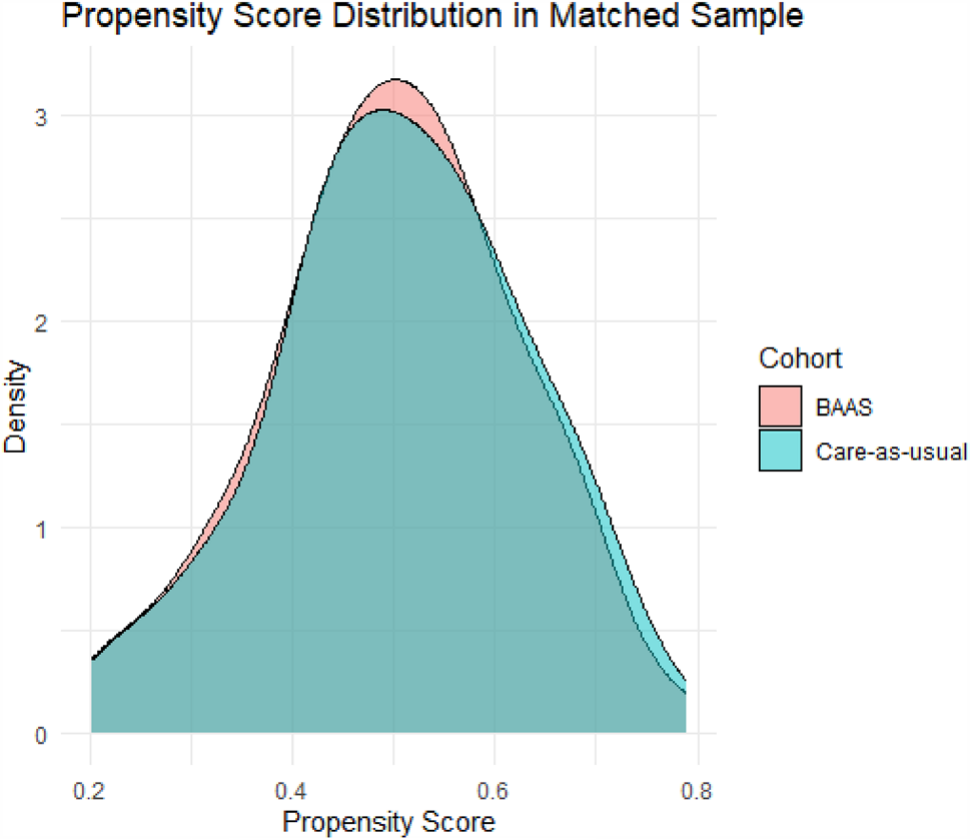
Propensity score density plot

A total of 270 patients (BAAS: *n* = 137, ACTIVE: *n* = 133) were included in this study (Fig. 1) between November 2021 and April 2024. Missing data on BMI (*n* = 4), preoperative sick leave (*n* = 3), presenteeism at 3/6/9/12 months post-surgery (*n* = 39/35/39/39), cost absenteeism (*n* = 8), and cost primary/secondary care (*n* = 40/40) were imputed in a total of 93 patients (BAAS *n* = 40, ACTIVE *n* = 53). Prior to matching, patients in the intervention and care-as-usual cohort did not differ significantly in age, sex, comorbidities, breadwinner, BMI, or preoperative sick leave. The cohorts did differ in level of education, type of surgery, and knee-straining jobs (Table 2). After matching, *n* = 102 were included for analysis per cohort. Covariate balance before and after matching was assessed using standardized mean differences and variance ratios, with thresholds of < 0.1 and < 2 respectively, indicating adequate balance across all covariates after matching (Appendix IV). Figure 2 shows substantial overlap of the propensity score distributions for the BAAS and care-as-usual cohorts, indicating that the matching procedure achieved good balance on the measured baseline covariates.

**Table 2:** Patient characteristics

	Before matching			After matching		
	BAAS (*n* = 137)	Care-as-usual (*n* = 133)	Difference (*p*)	BAAS (*n* = 102)	Care-as-usual (*n* = 102)	Difference (*p*)
Age (mean (SD))	58.5 (4.3)	58.7 (4.4)	0.581	58.1 (4.4)	58.9 (4.4)	0.232
Sex (%):			0.913			0.779
Female	73 (53.3)	69 (51.9)		54 (52.9)	57 (55.9)	
Male	64 (46.7)	64 (48.1)		48 (47.1)	45 (44.1)	
Education (%):			< 0.001			0.004
No	2 (1.5)	0 (0)		2 (2.0	0 (0.0)	
Primary	29 (21.2)	11 (8.3)		24(23.5)	11 (10.8)	
Lower secondary	72 (52.6)	59 (44.4)		50 (49.0)	48 (47.1)	
Higher secondary	12 (8.8)	9 (6.8)		7 (6.9)	5 (4.9)	
Higher vocational	18 (13.1)	48 (36.1)		15 (14.7)	34 (33.3)	
University or higher	1 (0.7)	6 (4.5)		1 (1.0)	4 (3.9)	
Comorbidities (%):			0.083			0.069
0	109 (72.6)	103 (77.4)		80 (78.4)	77 (75.5)	
1	20 (14.6)	20 (15.0)		15 (14.7)	16 (15.7)	
> 1	4 (2.9)	10 (7.5)		3 (2.9)	9 (8.8)	
Breadwinner (%):			0.105			0.104
Yes	58 (42.3)	52 (39.1)		44 (43.1)	41 (40.2)	
No	75 (54.7)	81 (60.9)		54 (52.9)	61 (59.8)	
Type of surgery			0.040			1.000
UKA	30 (21.9)	45 (33.8)		29 (28.4)	28 (27.5)	
TKA	107 (78.1)	88 (66.2)		73 (71.6)	74 (72.5)	
BMI (mean (SD))	29.2 (3.9)	29.7 (4.0)	0.293	29.3 (3.8)	29.4 (4.0)	0.801
Knee-straining job (%):			0.001			1.000
Yes	67 (48.9)	38 (28.6)		67 (65.7)	68 (66.7)	
No	70 (51.1)	95 (71.4)		35 (34.3)	34 (33.3)	
Sick leave before surgery (%):			0.134			0.865
Yes	30 (21.9)	27 (20.3)		81 (79.4)	79 (77.5)	
No	107 (78.1)	106 (79.7)		21 (20.6)	23 (22.5)

### Costs

The costs for the BAAS intervention were €845 per patient (movement sensor €60, occupational physician €128, occupational assessor €375, physical therapist hospital €242, and physical therapist primary care €40). Since no patients entered vocational rehabilitation, these costs were excluded. Differences in primary care (− €489) and absenteeism (− €7,263) were statistically significant, resulting in a significant difference from the employers and societal perspective of − €4493 and − €4982, respectively, in favor of the intervention group (Table 3). The distribution of costs is presented in Violin plots in Appendix III.

**Table 3:** Costs of the Back At work After Surgery care pathway versus care-as-usual

Costs	Costs BAAS [95% CI]	Costs CAU [95% CI]	Difference
Intervention BAAS	€845	–	€845
Medical care	€3,226 [2,917–3,535]	€3,715 [3,366–4,064]	€489 [26–953]
Primary	€1,407 [1,179–1,636]	€1,847 [1,551–2,143]	€440 [68–812]
Secondary	€1,819 [1,662–1,975]	€1,868 [1,745–1,991]	€49 [-149–247]
Productivity loss	€26,375 [23,923–28,827]	€31,713 [28,192–35,233]	€5,338 [1,070–9,605]
Absenteeism	€15,900 [13,895–17,905]	€23,163 [19,099–27,227]	€7,263 [2,749–11,777]
Presenteeism	€10,475 [8,986–11,964]	€8,550 [6,938–10,162]	€1,925 [-256 to 4,107]
Total employer’s costs	€27,220 [24,768–29,672]	€31,713 [28,192–35,233]	€4,493 [225–8,760]
Total societal costs	€30,446 [27,897–32,995]	€35,428 [31,792–39,065]	€4,982 [565–9,399]

### Return on Investment

The total netto benefits from employers and societal perspective were of €4,493 and €4,982, respectively, in the intervention cohort. The costs of the intervention were €845 per patient, resulting in an ROI of 590% [95% CI 67–1112%) from the societal perspective and 532% [95% CI 27–1037%] from the employers perspective per invested Euro (Table 3).

### Sensitivity Analysis

The first sensitivity analyses, using CART imputation, showed results still in favor of BAAS, though the differences were less pronounced. Specifically, difference in societal costs were − €4,430 [95% CI − 8,507 to − 353] compared to − €4,982 [95% CI − 9,399 to − 565] in the main analysis, and difference in productivity loss costs were − €3,991 [95% CI − 8,208 to 225] compared to − €5,338 [95% CI − 9,605 to − 1,70]. In the second sensitivity analysis, using a less optimal matching strategy (caliper of 0.2, resulting in *n* = 107 matched pairs), the results were similar, albeit slightly less pronounced: total societal costs difference of − €3,206 [95% CI − 7,369 to 958] and productivity loss costs difference of − €3,666 [95% CI − 7,686 to 355]. In the third sensitivity analysis, we repeated the matching and cost comparison in the complete case sample (*n* = 177). The BAAS group showed a cost difference of €2,814 (95% CI − €1,703 to €7,332, *p* = 0.220) in productivity loss and €2,409 (95% CI − €2,297 to €7,116, *p* = 0.313) in total societal cost compared to care-as-usual.

## Discussion

### Key Results

This study demonstrated that implementing the BAAS intervention yielded, on average, a positive financial return for both the employer (€4,493) and society (€4,982) at large. Notably, the return on investment (ROI) for the employer was statistically significant, indicating that for every Euro invested in the intervention, the employer made a profit of 532%. Interestingly, presenteeism costs were non-significantly higher in the BAAS group, which might be due to shorter absenteeism periods, causing patients to RTW earlier, but potentially with reduced productivity. This inverse relationship between absenteeism and presenteeism costs has also been noted in previous studies [32, 33].

### Comparison to Current Literature and Future Perspectives

Our findings align with existing literature highlighting the economic advantages of work-integrated interventions for musculoskeletal conditions. Work-integrated interventions after KA have demonstrated significant reductions in productivity loss, emphasizing the broader socioeconomic benefits of such strategies [34, 35]. Similar economic benefits have been observed in lumbar spine surgery, with reductions in absenteeism translating to substantial long-term savings [36]. Previous studies demonstrated the economic burden of knee osteoarthritis and KA, highlighting the importance of timely interventions [7, 37]. Abe et al. found that costs associated with non-operative treatments in the year before KA often exceed the costs incurred after surgery, indicating potential inefficiencies of prolonged non-operative management from an economic perspective [37]. However, our emphasis is not solely on earlier surgery, but rather on the timely and integrated coordination between medical and occupational care surrounding KA. Indeed, our findings show a positive return on investment mainly because the BAAS pathway resulted in 98% of patients successfully returning to full work following surgery instead of 13% in the care-as-usual cohort [20]. These results suggest an optimal balance between conservative management and timely surgical intervention, where work-related outcomes should be considered alongside clinical factors in decision-making. Similarly, our results are consistent with previous studies evaluating work-integrated interventions after KA. The Finnish Coordinated RTW (CRTW) program demonstrated positive effects on RTW, although the mean time to RTW (87 days) was notably longer than the median time in BAAS (27 days) [18, 19]. Although these outcomes cannot be perfectly compared (mean vs. median), the difference is notable. Moreover, 13% of CRTW participants did not RTW within one year, compared to only 2% in our BAAS cohort [18, 19]. These differences may reflect the more structured and interdisciplinary design of BAAS, which integrates occupational guidance earlier in the perioperative trajectory. Future research should focus on the long-term occupational and economic outcomes of work-integrated care interventions like the CRTW study or BAAS, including exploring sustainability beyond the initial postoperative year and the role of personalized, adaptive approaches in optimizing RTW trajectories after surgery.

### Strengths and Limitations

Our study had several strengths. First, this study included a large number of patients in two hospitals and eleven hospitals/clinics, increasing generalizability. Second, the application of nearest neighbor matching enhances the internal validity of our findings by adjusting for baseline differences. Also, sensitivity analyses showed results in the same direction. Lastly, the applied methods are in line with the Dutch guidelines for economic evaluations in healthcare, published by the National Health Care Institute of the Netherlands**,** ensuring methodological rigor and relevance for policy-making [27].

Our study had several limitations. First, presenteeism was measured using a questionnaire based on the IPCQ, wherein patients were asked about the number of days at work with reduced productivity over the past three months, which differs from reliably measuring reduced production at work [38]. Although measuring productivity remains subject to debate [39], presenteeism was assessed identically in both cohorts, minimizing systematic bias. Second, this study was conducted in the Netherlands, which may limit the generalizability of findings to other healthcare and workplace disability policies and reimbursement models. Third, the BAAS intervention cohort contained proportionally more participants with a lower level of education, even after matching (Table 2). Lower level of education has been linked to slower functional recovery and a longer time to RTW after KA [40–43]. Because education is highly correlated with physical job demands, we included only the “knee-straining job” variable in the propensity model to avoid multicollinearity, and this characteristic was balanced between groups. Residual confounding by education can therefore not be ruled out; if lower-educated workers indeed face a poorer prognosis, however, the current cost advantage observed for BAAS is likely an underestimate compared to a better education-balanced situation. In addition, patients with ≥ 2 comorbidities were over-represented in the care-as-usual cohort after matching, raising the possibility of residual confounding through multimorbidity-related cost inflation. Future studies should quantify the independent impact of educational level and multimorbidity on postoperative societal and employer costs after knee arthroplasty, to determine the extent of residual confounding in economic evaluations like ours. Fourth, our cost analysis did not include the one-off implementation expenses that hospitals incur when establishing a BAAS pathway from scratch—such as staff training, development of local protocols, and the purchase or leasing of monitoring devices (e.g., accelerometers). Although these start-up costs are non-recurring and diminish once BAAS is embedded, they would reduce the net financial gain in the first year of roll-out. Consequently, hospitals and clinics that are adopting BAAS de novo are unlikely to realize the reported ROI within their initial implementation year. In addition, workplace accommodations were advised by the occupational assessor if needed, but were not recorded in this study. As a result, this may have influenced productivity-related costs, potentially leading to an overestimation of the employer’s ROI. Lastly, we limited the follow-up to 12 months post-surgery. This one-year timeframe may be insufficient to capture the long-term sustainability of BAAS benefits. Any late-occurring costs or relapses into sick leave beyond the first year were not assessed, leaving uncertainty about the durability of the economic impact over multiple years. Because the BAAS cohort achieved a higher RTW rate than the care-as-usual cohort (98% vs. 87%), and Dutch employers must continue paying wages during the first two years of sick leave, the true employer cost differential is likely larger than the estimate reported in this study [20]. Moreover, as demonstrated by Zaballa et al., some patients who have KA initially successfully RTW but subsequently exit employment due to persistent knee-related problems, underscoring the importance of monitoring longer-term occupational outcomes [44].

## Conclusion

By combining medical and occupational care, the BAAS work-integrated care pathway reduces overall costs, costs of productivity loss, and medical costs after KA, yielding a positive ROI from employers and societal perspective.

## Supplementary Information

Below is the link to the electronic supplementary material.

Supplementary file1 (DOCX 738 KB)

## Data Availability

The datasets generated during and/or analyzed during the current study are available from the corresponding author on reasonable request.
