# Supplementary material for: Return on Investment of the Back At Work After Surgery (BAAS) Care Pathway Compared to Care-as-Usual in Knee Arthroplasty
Source: J Occup Rehabil. 2025 Sep 19;35(4):958–66. doi: 10.1007/s10926-025-10328-w (PMC12575464; doi:10.1007/s10926-025-10328-w)

Appendix I


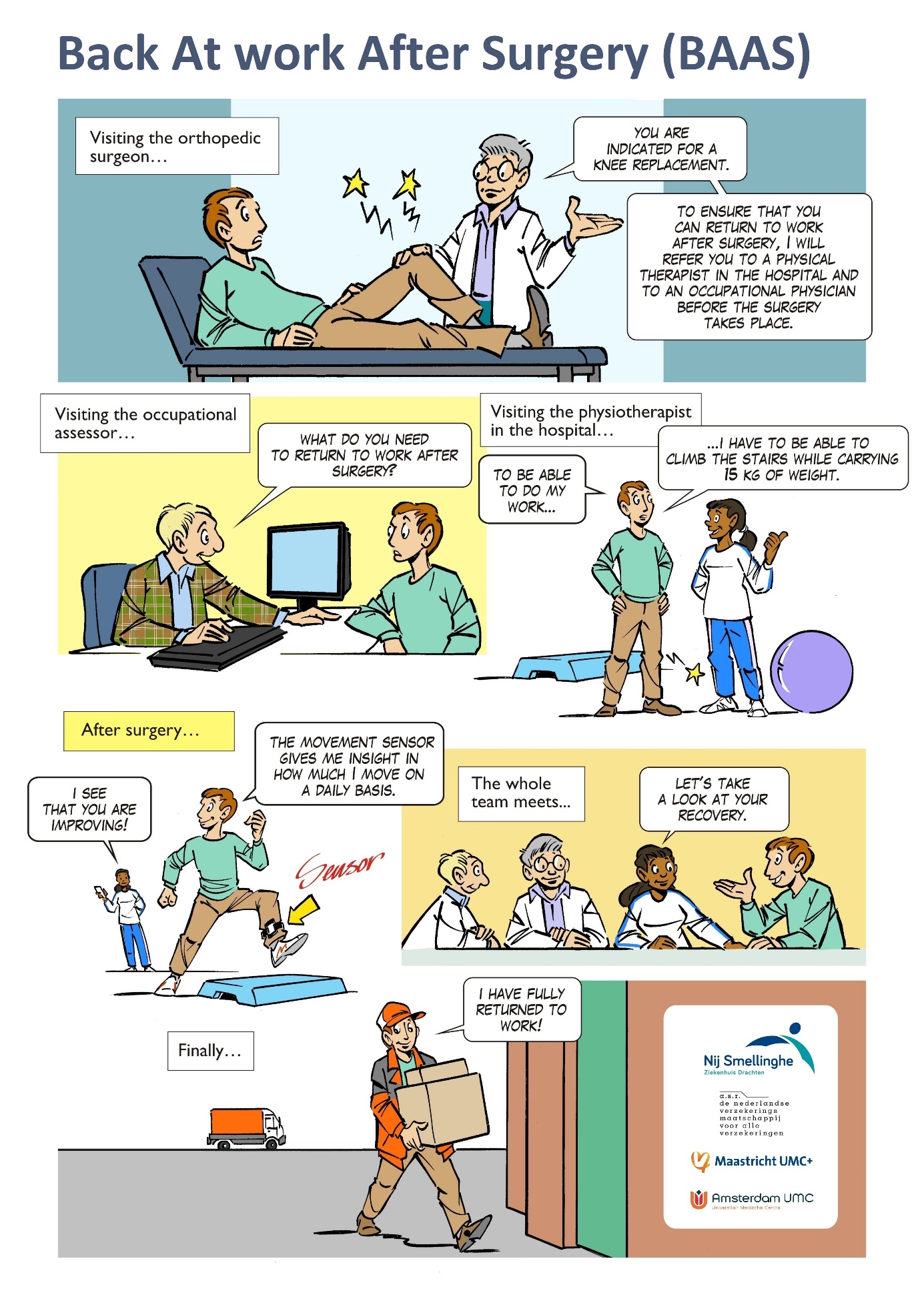


Appendix II – R Script

##############################################

# Script for Publication: SPSS Data Analysis,

# Multiple Imputation, Matching, and Cost Analysis

##############################################

# Load required libraries

library(haven)

library(mice)

library(dplyr)

library(tidyr)

library(ggplot2)

library(tableone)

library(cobalt)

library(MatchIt)

library(patchwork)

##############################################

# 1. Data Import and Preparation

##############################################

# Read the SPSS data file

data <- read_sav("database.sav")

# Convert selected variables to factors

data$Sex <- as.factor(as_factor(data$Sex))

data$Education <- as.factor(as_factor(data$Education))

data$Comorbidities <- as.factor(as_factor(data$Comorbidities))

data$Breadwinner <- as.factor(as_factor(data$Breadwinner))

data$Type_Operation <- as.factor(as_factor(data$Type_Operation))

data$Knee_straining_work <- as.factor(as_factor(data$Knee_straining_work))

data$Sick_leave <- as.factor(as_factor(data$Sick_leave))

data$Relation_Kneecompaints_Work <- as.factor(as_factor(data$Relation_Kneecompaints_Work))

# Replace value 999 with NA in selected variables

vars_to_impute <- c(

"Sex", "Type_Operation", "BMI", "Knee_straining_work", "Sick_leave",

"Relation_Kneecompaints_Work",

"WAS_T1", "WAS_T2", "WAS_T3", "WAS_T4",

"Cost_if_sickleave_allyear", "Cost_Absenteeism",

"Cost_Primary_Care", "Cost_Secundairy_Care"

)

data[vars_to_impute] <- lapply(data[vars_to_impute], function(x) ifelse(x == 999, NA, x))

# Optional: check missingness before imputation

missing_before <- sapply(data[vars_to_impute], function(x) sum(is.na(x)))

cat("Missing values per variable before imputation:\n")

print(missing_before)

# Prepare data for imputation

impute_vars <- c(vars_to_impute, "Cohort", "RTW_full_Days")

data_imp <- data[, impute_vars]

##############################################

# 2. Multiple Imputation using PMM

##############################################

imp_pmm <- mice(data_imp, m = 5, seed = 123, method = "pmm")

# Display imputation information

imputation_counts <- sapply(imp_pmm$imp, nrow)

cat("Number of imputations per variable (PMM):\n")

print(imputation_counts)

data_imp$had_imputation <- apply(data_imp[, vars_to_impute], 1, function(x) any(is.na(x)))

patients_imputed_per_cohort <- table(data_imp$Cohort, data_imp$had_imputation)

cat("Patients per Cohort with imputation (TRUE = at least one):\n")

print(patients_imputed_per_cohort)

##############################################

# 3. Cost Computation and Aggregation

##############################################

compute_costs <- function(df) {

df %>% mutate(

WAS_mean_full = case_when(

RTW_full_Days <= 91 ~ rowMeans(select(., WAS_T1, WAS_T2, WAS_T3, WAS_T4), na.rm = TRUE),

RTW_full_Days <= 182 ~ rowMeans(select(., WAS_T2, WAS_T3, WAS_T4), na.rm = TRUE),

RTW_full_Days <= 273 ~ rowMeans(select(., WAS_T3, WAS_T4), na.rm = TRUE),

RTW_full_Days <= 364 ~ rowMeans(select(., WAS_T4), na.rm = TRUE),

TRUE ~ 10

),

Cost_Presenteeism = (Cost_if_sickleave_allyear - Cost_Absenteeism) * (1 - (WAS_mean_full / 10)),

Productivity_loss = Cost_Absenteeism + Cost_Presenteeism,

Medical_cost = Cost_Primary_Care + Cost_Secundairy_Care

)

}

##############################################

# 4. Unweighted Means and 95% Confidence Intervals per Cohort

##############################################

cost_vars <- c(

"Cost_Absenteeism", "Cost_Primary_Care", "Cost_Secundairy_Care",

"Cost_Presenteeism", "Productivity_loss", "Medical_cost"

)

mean_ci <- function(x) {

m <- mean(x, na.rm = TRUE)

s <- sd(x, na.rm = TRUE)

n <- sum(!is.na(x))

error <- qt(0.975, df = n - 1) * s / sqrt(n)

c(mean = m, lower = m - error, upper = m + error)

}

complete_data_pmm <- complete(imp_pmm, 1) %>%

compute_costs() %>%

mutate(Cohort = factor(Cohort))

ci_stats <- complete_data_pmm %>%

group_by(Cohort) %>%

summarise(across(all_of(cost_vars), ~ list(mean_ci(.)))) %>%

pivot_longer(cols = all_of(cost_vars), names_to = "Variable", values_to = "Stats") %>%

unnest_wider(Stats)

cat("Unweighted means and 95% CI per Cohort (PMM):\n")

print(ci_stats)

##############################################

# 4.5 Table 1: Baseline Descriptives with p‑values

##############################################

# Add baseline vars (Age, Education, Comorbidities, Breadwinner) from original data

complete_data_pmm <- complete_data_pmm %>%

mutate(.row = row_number()) %>%

left_join(

data %>% mutate(.row = row_number()) %>%

select(.row, Age, Education, Comorbidities, Breadwinner),

by = ".row"

) %>%

select(-.row)

vars_table1 <- c(

"Age", "Sex", "Education", "Comorbidities", "Breadwinner",

"Type_Operation", "BMI", "Knee_straining_work", "Sick_leave"

)

cat_vars <- c(

"Sex", "Education", "Comorbidities", "Breadwinner",

"Type_Operation", "Knee_straining_work", "Sick_leave"

)

table1 <- CreateTableOne(

vars = vars_table1,

strata = "Cohort",

data = complete_data_pmm,

factorVars = cat_vars

)

print(

table1,

showAllLevels = TRUE,

test = TRUE,

formatOptions = list(contDigits = 2)

)

##############################################

# 5. Outcome Analysis via Linear Models (Pooling with Rubin's Rule)

##############################################

pool_results_list <- lapply(cost_vars[1:4], function(var) {

form <- as.formula(paste(var, "~ Cohort"))

fit <- with(imp_pmm, {

tmp <- data.frame(

RTW_full_Days = RTW_full_Days,

WAS_T1 = WAS_T1,

WAS_T2 = WAS_T2,

WAS_T3 = WAS_T3,

WAS_T4 = WAS_T4,

Cost_if_sickleave_allyear = Cost_if_sickleave_allyear,

Cost_Absenteeism = Cost_Absenteeism,

Cost_Primary_Care = Cost_Primary_Care,

Cost_Secundairy_Care = Cost_Secundairy_Care,

Cohort = Cohort

)

tmp <- compute_costs(tmp)

lm(form, data = tmp)

})

pooled <- pool(fit)

pooled_summary <- summary(pooled, conf.int = TRUE)

diff_row <- pooled_summary[grep("Cohort", pooled_summary$term), ]

diff_row$Variable <- var

diff_row

})

diff_stats <- do.call(rbind, pool_results_list)

cat("Difference in mean costs between Cohorts (pooled via Rubin's Rule, PMM):\n")

print(diff_stats)

##############################################

# 6. Nearest Neighbor Matching

##############################################

matched_data <- complete_data_pmm %>%

mutate(Cohort = recode(Cohort, `1` = "BAAS", `2` = "Care-as-usual"))

m.out <- matchit(

Cohort ~ Sex + Type_Operation + BMI + Knee_straining_work +

Sick_leave + Relation_Kneecompaints_Work,

data = matched_data,

method = "nearest",

caliper = 0.1

)

matched_data <- match.data(m.out)

cat("Number of matched individuals per group:\n")

print(table(matched_data$Cohort))

matched_data <- matched_data %>%

mutate(

Total_cost = ifelse(

Cohort == "BAAS",

Productivity_loss + Medical_cost + 845,

Productivity_loss + Medical_cost

)

)

##############################################

# 6.5 Table 1: Baseline Descriptives in Matched Sample

##############################################

# Variabelen die je in Table.1 wilt zien

vars_table1_matched <- c(

"Age", "Sex", "Education", "Comorbidities", "Breadwinner",

"Type_Operation", "BMI", "Knee_straining_work", "Sick_leave"

)

# Geef aan welke variabelen categorisch zijn

cat_vars_matched <- c(

"Sex", "Education", "Comorbidities", "Breadwinner",

"Type_Operation", "Knee_straining_work", "Sick_leave"

)

# Maak Table 1 voor de matched sample

table1_matched <- CreateTableOne(

vars = vars_table1_matched,

strata = "Cohort",

data = matched_data,

factorVars = cat_vars_matched

)

# Print met mean (SD) voor continue, n(%) voor categorisch, én p‑waarden

print(

table1_matched,

showAllLevels = TRUE,

test = TRUE,

formatOptions = list(contDigits = 2)

)

##############################################

# 6.7 Covariate Balance Diagnostics with cobalt

##############################################

# Create balance object to evaluate covariate balance before and after matching

bal.tab.out <- bal.tab(

m.out,

un = TRUE, # Show balance before matching

m.threshold = 0.1, # Threshold for standardized mean difference (SMD)

v.threshold = 2 # Threshold for variance ratio (VR)

)

# Print numeric summary of balance (SMDs and variance ratios)

cat("Covariate Balance Diagnostics:\n")

print(bal.tab.out)

# Generate a Love plot to visually display covariate balance

love.plot(

m.out,

threshold = 0.1, # Dashed vertical line at 0.1 for SMD

var.order = "unadjusted", # Order variables by unadjusted SMD

abs = TRUE, # Use absolute values of SMDs

colors = c("gray50", "black")

) +

ggtitle("Standardized Mean Differences Before and After Matching")

##############################################

# 7. Outcome Table for Matched Sample

##############################################

final_cost_vars <- c(cost_vars, "Total_cost")

final_table_matched <- data.frame(

Costs = character(),

`Costs BAAS [95% CI]` = character(),

`Costs CAU [95% CI]` = character(),

Difference = character(),

p_value = numeric(),

stringsAsFactors = FALSE

)

for (var in final_cost_vars) {

g_b <- matched_data %>% filter(Cohort == "BAAS") %>% pull(!!sym(var))

g_c <- matched_data %>% filter(Cohort == "Care-as-usual") %>% pull(!!sym(var))

m_b <- mean(g_b, na.rm = TRUE); s_b <- sd(g_b, na.rm = TRUE); n_b <- sum(!is.na(g_b))

m_c <- mean(g_c, na.rm = TRUE); s_c <- sd(g_c, na.rm = TRUE); n_c <- sum(!is.na(g_c))

ci_b <- qt(0.975, df = n_b - 1) * s_b / sqrt(n_b)

ci_c <- qt(0.975, df = n_c - 1) * s_c / sqrt(n_c)

t_res <- t.test(g_c, g_b)

diff_val <- m_c - m_b

baas_str <- sprintf("€%.0f [%.0f–%.0f]", m_b, m_b - ci_b, m_b + ci_b)

cau_str <- sprintf("€%.0f [%.0f–%.0f]", m_c, m_c - ci_c, m_c + ci_c)

diff_str <- sprintf("€%.0f [%.0f–%.0f]", diff_val, t_res$conf.int[1], t_res$conf.int[2])

final_table_matched <- rbind(

final_table_matched,

data.frame(

Costs = var,

`Costs BAAS [95% CI]` = baas_str,

`Costs CAU [95% CI]` = cau_str,

Difference = diff_str,

p_value = t_res$p.value,

stringsAsFactors = FALSE

)

)

}

final_table_matched$Costs <- recode(

final_table_matched$Costs,

Cost_Primary_Care = "Medical Care – Primary",

Cost_Secundairy_Care = "Medical Care – Secondary",

Cost_Absenteeism = "Productivity Loss – Absenteeism",

Cost_Presenteeism = "Productivity Loss – Presenteeism",

Productivity_loss = "Productivity Loss",

Medical_cost = "Medical Cost",

Total_cost = "Total Cost"

)

cat("Final Outcome Table for Matched Sample:\n")

print(final_table_matched)

##############################################

# 8. Diagnostic Plot: Propensity Score Distribution

##############################################

diagnostic_plot <- ggplot(matched_data, aes(x = distance, fill = Cohort)) +

geom_density(alpha = 0.5) +

labs(

title = "Propensity Score Distribution in Matched Sample",

x = "Propensity Score", y = "Density"

) +

theme_minimal()

print(diagnostic_plot)

##############################################

# 9. Combined Violin Plot for Cost Components

##############################################

outcome_vars <- c(

"Cost_Absenteeism", "Cost_Presenteeism",

"Cost_Primary_Care", "Cost_Secundairy_Care"

)

violin_data <- matched_data %>%

select(Cohort, all_of(outcome_vars)) %>%

pivot_longer(

cols = all_of(outcome_vars),

names_to = "Outcome",

values_to = "CostValue"

) %>%

mutate(Outcome = recode(

Outcome,

Cost_Absenteeism = "Absenteeism",

Cost_Presenteeism = "Presenteeism",

Cost_Primary_Care = "Primary care",

Cost_Secundairy_Care = "Secondary care"

))

fill_colors <- c("BAAS" = "blue", "Care-as-usual" = "red")

violin_plot <- ggplot(violin_data, aes(x = Cohort, y = CostValue, fill = Cohort)) +

geom_violin(trim = FALSE, scale = "width", adjust = 1) +

facet_wrap(~Outcome, scales = "free_y") +

scale_fill_manual(values = fill_colors) +

labs(x = "", y = "Cost (€)") +

scale_y_continuous(limits = c(0, NA)) +

theme_minimal() +

theme(

axis.text.x = element_blank(),

axis.ticks.x = element_blank(),

strip.text = element_text(size = 12)

)

print(violin_plot)

##############################################

# 10. Calculate ROI for Productivity Loss and Total Cost (Matched Sample, PMM)

##############################################

# Productivity Loss ROI: (difference – 845) / 845

prod_b <- matched_data %>% filter(Cohort == "BAAS") %>% pull(Productivity_loss)

prod_c <- matched_data %>% filter(Cohort == "Care-as-usual") %>% pull(Productivity_loss)

t_prod <- t.test(prod_c, prod_b)

diff_prod <- mean(prod_c, na.rm = TRUE) - mean(prod_b, na.rm = TRUE)

roi_prod <- (diff_prod - 845) / 845

roi_prod_lower <- (t_prod$conf.int[1] - 845) / 845

roi_prod_upper <- (t_prod$conf.int[2] - 845) / 845

# Total Cost ROI (unchanged)

tot_b <- matched_data %>% filter(Cohort == "BAAS") %>% pull(Total_cost)

tot_c <- matched_data %>% filter(Cohort == "Care-as-usual") %>% pull(Total_cost)

t_tot <- t.test(tot_c, tot_b)

diff_tot <- mean(tot_c, na.rm = TRUE) - mean(tot_b, na.rm = TRUE)

roi_tot <- diff_tot / 845

roi_tot_lower <- t_tot$conf.int[1] / 845

roi_tot_upper <- t_tot$conf.int[2] / 845

roi_table <- data.frame(

Outcome = c("Productivity Loss", "Total Cost"),

ROI = c(round(roi_prod, 2), round(roi_tot, 2)),

ROI_lower = c(round(roi_prod_lower, 2), round(roi_tot_lower, 2)),

ROI_upper = c(round(roi_prod_upper, 2), round(roi_tot_upper, 2)),

stringsAsFactors = FALSE

)

cat("\nROI for Productivity Loss and Total Cost in Matched Sample (PMM):\n")

print(roi_table)

##############################################

# 11. Sensitivity Analysis: Complete Case Matching and Cost Differences

##############################################

# Select complete cases on relevant variables

vars_needed_cc <- c(

"Cohort", "Sex", "Type_Operation", "BMI", "Knee_straining_work",

"Sick_leave", "Relation_Kneecompaints_Work",

"WAS_T1", "WAS_T2", "WAS_T3", "WAS_T4",

"Cost_if_sickleave_allyear", "Cost_Absenteeism",

"Cost_Primary_Care", "Cost_Secundairy_Care",

"RTW_full_Days"

)

complete_data_cc <- data %>%

select(all_of(vars_needed_cc)) %>%

filter(if_all(everything(), ~ !is.na(.)))

cat("Number of complete cases per cohort:\n")

print(table(complete_data_cc$Cohort))

# Compute costs

complete_data_cc <- compute_costs(complete_data_cc)

# Ensure Cohort is a factor with numeric labels (1 and 2)

complete_data_cc$Cohort <- factor(complete_data_cc$Cohort, levels = c(1, 2))

# Perform matching on complete cases

m.out.cc <- matchit(

Cohort ~ Sex + Type_Operation + BMI + Knee_straining_work +

Sick_leave + Relation_Kneecompaints_Work,

data = complete_data_cc,

method = "nearest",

caliper = 0.1

)

# Extract matched data

matched_data_cc <- match.data(m.out.cc) %>%

mutate(

Cohort = recode(as.character(Cohort), `1` = "BAAS", `2` = "Care-as-usual"),

Total_cost = ifelse(

Cohort == "BAAS",

Productivity_loss + Medical_cost + 845,

Productivity_loss + Medical_cost

)

)

# Calculate mean differences and 95% CI for Productivity Loss

prod_b_cc <- matched_data_cc %>% filter(Cohort == "BAAS") %>% pull(Productivity_loss)

prod_c_cc <- matched_data_cc %>% filter(Cohort == "Care-as-usual") %>% pull(Productivity_loss)

t_prod_cc <- t.test(prod_c_cc, prod_b_cc)

diff_prod_cc <- mean(prod_c_cc, na.rm = TRUE) - mean(prod_b_cc, na.rm = TRUE)

# Calculate mean differences and 95% CI for Total Cost

tot_b_cc <- matched_data_cc %>% filter(Cohort == "BAAS") %>% pull(Total_cost)

tot_c_cc <- matched_data_cc %>% filter(Cohort == "Care-as-usual") %>% pull(Total_cost)

t_tot_cc <- t.test(tot_c_cc, tot_b_cc)

diff_tot_cc <- mean(tot_c_cc, na.rm = TRUE) - mean(tot_b_cc, na.rm = TRUE)

# Create result table

cost_diff_table_cc <- data.frame(

Outcome = c("Productivity Loss (CC)", "Total Cost (CC)"),

Difference = c(round(diff_prod_cc, 0), round(diff_tot_cc, 0)),

Lower_CI = c(round(t_prod_cc$conf.int[1], 0), round(t_tot_cc$conf.int[1], 0)),

Upper_CI = c(round(t_prod_cc$conf.int[2], 0), round(t_tot_cc$conf.int[2], 0)),

p_value = c(signif(t_prod_cc$p.value, 3), signif(t_tot_cc$p.value, 3)),

stringsAsFactors = FALSE

)

cat("\nSensitivity Analysis – Cost Differences in Complete Cases:\n")

print(cost_diff_table_cc)

Appendix III - Cost distribution of absenteeism, presenteeism, primary and secondary medical care. Note the varying y-axis.


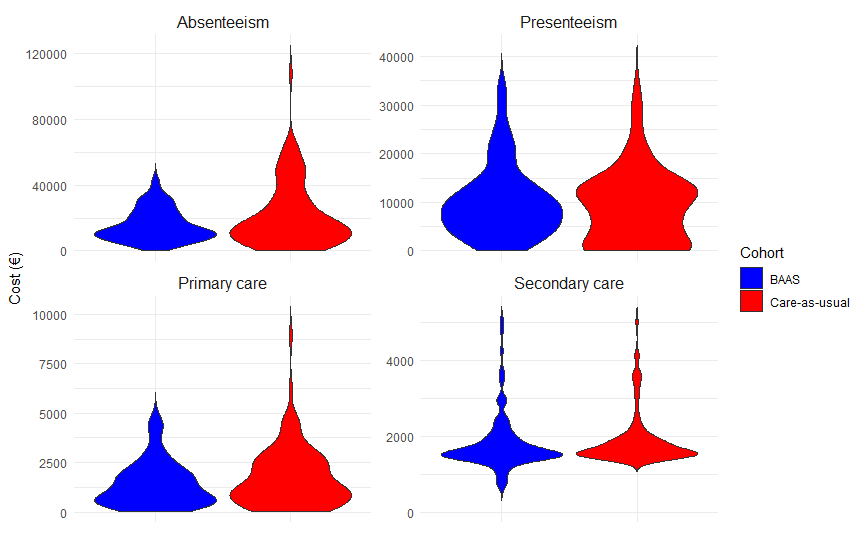


Appendix IV – Love plot of standardized mean differences before and after matching


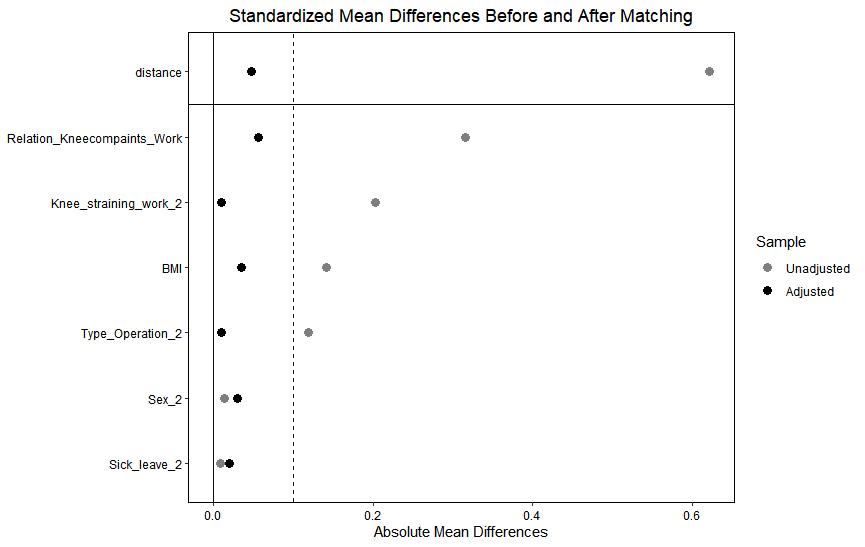

Supplement: Supplementary file 1 — Supplementary file1 (DOCX 738 KB) [file 10926_2025_10328_MOESM1_ESM.docx]
